# Microbial Pretreatment of Chicken Feather and Its Co-digestion With Rice Husk and Green Grocery Waste for Enhanced Biogas Production

**DOI:** 10.3389/fmicb.2022.792426

**Published:** 2022-04-07

**Authors:** Marium Saba, Anum Khan, Huma Ali, Amna Bibi, Zeeshan Gul, Alam Khan, Muhammad Maqsood Ur Rehman, Malik Badshah, Fariha Hasan, Aamer Ali Shah, Samiullah Khan

**Affiliations:** ^1^Department of Microbiology, Faculty of Biological Sciences, Quaid-i-Azam University, Islamabad, Pakistan; ^2^State Key Laboratory, Grassland Argo-Ecosystem, School of Life Sciences, Lanzhou University, Lanzhou, China

**Keywords:** anaerobic digestion, biogas, *Pseudomonas aeruginosa*, microbial pretreatment, chicken feather waste

## Abstract

To utilize wastes and residues sustainably and excellently, there is a need to fend for efficient methods and resources for biogas production. Use of poultry waste for biogas production represents one of the most important routes toward reaching global renewable energy targets. The current study involves microbial pretreatment of chicken feather waste, followed by its co-digestion with rice husk and green grocery waste in batch and continuous reactors, respectively. Microbial pretreatment of chicken feathers by keratinase secreting *Pseudomonas aeruginosa* was an effective and eco-friendly approach to make its recalcitrant structure available as a raw substrate for biogas production. The current study also addressed the enhancement and stability of anaerobic digestion by co-digestion. Results demonstrated that biogas production was increased by microbial pretreatment of chicken feathers and that the percentage increase in biogas yield was 1.1% in microbialy pretreated feathers compared to mono-digestion (non-pretreated feathers) in batch fermentation. The highest yield of biogas was obtained in a batch reactor having co-digestion of pretreated rice husk and microbial pretreated chicken feathers. The co-digestion of chicken feathers hydrolysate with green grocery waste in continuous fermentation mode has also enhanced the biogas yield as compared to average of mono-digestion (chicken feather hydrolysate and green grocery waste) and, therefore, improve the efficiency of the overall process.

## Introduction

Bioenergy may be a significant replacement for non-renewable fossil fuels, making the path easier for sustainable development and decreasing the dependency on conventional energy sources. The world population is increasing rapidly; industrial activities are flourishing due to it. Global urbanization and industrialization lead to enhanced energy demands and more waste generation (Zhang et al., 2021). Moreover, as energy is the critical source of comfort for the modern world, most developing and underdeveloped countries have no proper access to energy services like petrol, diesel, natural gas, and electricity. Fossil fuels are the primary energy source for many countries, but their reservoirs will be scarce to meet requirements in the near future. Scientists are looking for renewable sources to overcome fuels limitations (Forgács, 2012; Gooding and Meeker, 2016).

On the other hand, globally, 17 billion tons of waste per annum is being generated due to increased industrialization and urbanization; it will reach 27 billion tons by 2050 (Patinvoh et al., 2016; Rahaman et al., 2021). Biogas is an emerging renewable energy source obtained through the degradation of organic matter by microorganisms under anaerobic conditions. The biogas production and applications provide a comprehensive and systematic guide to developing and deploying biogas supply chains and technology (Ogut et al., 2013; Kanafin et al., 2021).

According to the world’s food demands, the poultry industry is major growing strength. The necessity to cope with waste disposal needs some severe alternatives; otherwise, it will lead to global and environmental pollution (Morinval and Averous, 2021). Millions of tons of organic waste are produced annually from the poultry industry (Karuppannan et al., 2021). These wastes have high capabilities to generate methane. The poultry industry generates organic waste like feathers in a vast quantity. Feathers are composed of keratin protein, having a recalcitrant structure. The cross-linking and covalent bonds added more strength to the feather’s structure (Sypka et al., 2021). Different pretreatment methods are used like superheated water, strong acids, or alkalis puffing, and pressurized breakdown of feathers (Chao et al., 2021), and many other physiochemical methods could be applied (Wang et al., 2012, 2019).

Because of high cost, sustainability, and ecological problems, these pretreatment methods are seldom used. Recent research trends in cost-effective, ecological, and sustainable pretreatment methods with the help of microbial consortia (González et al., 2021). Different microorganisms like bacteria and fungi were isolated for pretreatment of chicken feathers (CFs). CFs were biologically pretreated, and the pretreated feathers hydrolysate was used for biogas production. Production of biogas *via* an anaerobic digestion (AD) process effectively reduced the waste generated in large volumes. It is a sustainable and well-established process to utilize waste for energy production. The digestate that is leftover could be still used as a source of fertilizers (Awasthi et al., 2022).

AD is a multistep, sensitive, and complex process with different microorganisms (Hagos et al., 2017). This process occurs in an oxygen-deficient environment. Methane and CO_2_ are produced from a chain of different biochemical processes during AD. AD has four different stages: hydrolysis, acidogenesis, acetogenesis, and methanogenesis (Peng et al., 2021), in which the organic substances are broken down in the absence of oxygen. The organic matter that is not digested in the AD process is considered digestate. The vital operating parameters that must be maintained and optimized for an AD process are proper carbon-to-nitrogen (C/N) ratio, pH, temperature, total solid (TS) contents, and volatile solid (VS) contents (Ellacuriaga et al., 2021). Pretreatment is essential to improve the biogas production and process stability. Therefore, it will be helpful to investigate the optimal mix of substrates and the conditions of fermentation. Biological pretreatment can be carried out either by fungi or bacteria to degrade keratin waste. Bacteria use keratin as a source of carbon and energy (Chaturvedi et al., 2021). However, high nitrogen content in CFs with a low C/N ratio will not suit AD. Therefore, the feathers, a protein-rich substrate, must be co-digested with another substrate with high carbon content to balance the C/N ratio (Guo et al., 2012; Patinvoh et al., 2017; Mohanty et al., 2021). The rate of AD is affected significantly by the C/N ratio. A balance C/N ratio is required for microorganisms’ growth and proper metabolism. The reported C/N ratio for proper digestion is 20–30:1 (Zheng et al., 2021). The biomethane potential of many wastes in batch fermentation experiments has been tested, and few studies compare batch fermentation to continuous fermentation. Batch fermentations are suitable for estimating a substrate’s methane yield and biodegradability, but they do not provide information about the long-term effect of the substrate on the fermentation (Szilágyi et al., 2021).

CF contributes a significant portion to poultry waste. High content of protein in CF waste is associated with the problem of ammonia inhibition. Therefore, to address this problem, the present study was designed to increase the biogas production from CFs by biological pretreatment by *Pseudomonas aeruginosa* and co-digestion with carbon-rich rice husk (RH) and green grocery waste (GW) to avoid ammonia inhibition. It will help to improve the feather waste to energy conversion through the AD process.

## Materials and Methods

### Substrates for Anaerobic Digestion

Different substrates used during the current study were CF, CFs’ hydrolysate (HL), rice husk (RH), and green grocery waste (GW). The CF was collected from a slaughterhouse (a poultry shop in Quaid-i-Azam university, Islamabad, Pakistan) and, after collection, the feathers were washed with tap water to remove blood and mud attached to it. After that, it was sun-dried for 3 days and was cut into small pieces manually to increase the surface area for microbial pretreatment. Pakistan is an agricultural country, where rice is abundantly cultivated; therefore, RH was used as a co-substrate in AD and was collected from Swat (District in the Malakand Division of Khyber Pakhtunkhwa, Pakistan) stones, and other solid particles present in the RH were removed. It was then chopped in a grinder to increase its surface area. The GW was purchased from juice shops of Quaid-i-Azam University, Islamabad, Pakistan, and used as a co-substrate in continuous AD. Solid particles present in the GW were removed. It was then chopped in a grinder to increase its surface area. The CF and RH were stored at room temperature, whereas GW was stored in plastic bags at −20°C until further use. Weighed substrates were thoroughly mixed during the experiment to gain a homogenized feed before use.

### Isolation and Screening of Microorganisms for Pretreatment

For keratinase production, sample of soil was collected from the dumping site in poultry shop. Serial dilution was performed up to 10 test tubes. A 0.1 ml of diluted suspension was spread over an agar plate, to get isolated colonies. The isolated colonies were then further purified to get pure colonies of microorganisms. Keratinase screening was carried by spreading the pure colonies over skim milk agar medium and analyzed after 24 h for caseinolytic activity of the microorganisms. Those strains that produce clear zones of hydrolysis were selected. Among them, C1 was selected on the basis of maximum specific activity (1.24 U/mg) and its feather hydrolyzing ability within 48 h of incubation. The isolate was identified as *Pseudomonas aeruginosa* strain C1M (95.30% 16S rRNA gene identity with *Pseudomonas aeruginosa* 1MP68).

### Pretreatment of Chicken Feathers and Rice Husk

For biogas production, the CFs were biologically pretreated with keratinase-producing microorganisms, i.e., *Pseudomonas aeruginosa*, to increase its biodegradability prior to AD. For microbial pretreatment of CFs (substrate), feather basal medium was prepared with composition NaCl (0.5 g/L), Na_2_SO_4_ (0.5 g/L), KH_2_PO_4_ (0.4 g/L), and K_2_HPO_4_ (0.3 g/L) with dried feathers 1, 2, or 3% to the flask of medium autoclaved at 121°C for 20 min. Two to three milliliters of inoculum per 100 ml of the feather basal medium were added and incubated for 5–8 days at 37°C and 150 rpm. The negative control without the addition of microorganisms was run in parallel. The degraded feather–containing medium was designated as whole broth or hydrolysate of CFs and stored at −20°C until further use.

Prior to biogas production, RH (1.59 g VS) was pretreated with 1% phosphoric acid and autoclaved at 121°C for 20 min.

### Substrate Characterization for Anaerobic Digestion

The CFs, microbial pretreated CFs’ hydrolysate, RH, and GW were used as the substrate for AD.

The TSs and VSs in all the substrates used in the experiments were determined according to National Renewable Energy Laboratory’s analytical procedures. The usual procedures were used to determine the sample’s TS and VS (Pinheiro et al., 2021), and the analysis of each sample was performed in triplicate. The characteristics of the substrates are demonstrated in Table 1.

**TABLE 1:** Characteristics of substrate and inoculum.

Biomass	TS (%)	VS of TS (%)
Chicken feather	83.86 ± 7.98 a	83.47 ± 8.28 a
Chicken feather hydrolysate (Microbial pretreated)	1.36 ± 0.08 b	77.16 ± 5.11 ab
Rice husk	91.36 ± 8.47 a	83.17 ± 5.97 a
Inoculum	1.18 ± 0.09 c	68.10 ± 6.43 b

*Means having the same letter are statistically similar as per the Tukey test at p < 0.05.*

### Inoculum Development

The inoculum used for the batch fermentation was collected from the running experimental biogas reactor at Sustainable Bioenergy and Biorefinery Laboratory, Department of Microbiology, Quaid-i-Azam University. The operational conditions of the reactors were retention time 10 days, organic loading rates 3 g, co-digestion of cattle manure with fruits and vegetables, and two-stage AD process waste. The inoculum was developed and degassed in a 3-L volume of anaerobic reactors. The reactors were incubated at 37°C for 20 days to allow the microbes to eliminate any organic matter in the inoculum. The inoculum for continuous anaerobic fermentation was acquired from the National Agriculture Research Council’s running experimental biogas reactor. The retention time of the digester was 57 days, and it was kept at 37°C. The inoculum was said to be fully developed when it starts biogas production. The inoculum could be used in experiments when there is slowly decrease in biogas production was observed. The VS and TS were determined as previously explained and presented in Table 1.

### Anaerobic Digestion

The present study was conducted in batch fermentation, at 37°C, to determine potential of CFs, RH, and the effect of pretreatment and co-digestion on the biogas production. Continuous fermentation at 37°C was also performed to determine the biogas potential of CFs’ hydrolysate with GW in co-digestion and also separately in mono-digestion.

#### Experimental Setups for Batch Anaerobic Digestion

The experimental setup for the biogas production during the batch process was carried out in 500-ml glass reactors with a working volume of 400 ml, presented in Table 2. In the present study, eight different setups were designed for 60 days, and each setup was carried out in a triplicate. The positive and negative controls were run at the same conditions in parallel. The negative control contained only inoculum to find out the amount of biogas production in the background of the test reactors. The positive control (PC) containing cellulose ensures the inoculum activity in the reactors. In the third reactor, the feasibility of non-pretreated feathers for biogas production was also studied. Dried non-pretreated feathers were cut down into a small size, and then, a specific amount was added to the reactor. In this reactor, the feather is supposed to be the substrate. The fourth reactor contains hydrolysate, microbial pretreated CFs, as a substrate. The fifth reactor contains a non-pretreated RH (RH-P) in a powder form, whereas the other reactor has a RH-P with inoculum. The pretreated and non-pretreated reactors were used to check the impact of pretreatment over non-pretreatment biogas production. The seventh reactor contains the hydrolysate and its co-digestion with non–RH-P. The last setup has microbial pretreated CFs’ hydrolysate with RH-P. The inoculum and substrate were added in the reactors in a ratio of 4:1 in terms of VS, and the amount of substrate required was calculated from the formula given below. The inoculum was added to the substrate to make a working volume of 400 ml.


$$\mathrm{Amount}\,\mathrm{of}\,\mathrm{substrate}=\frac{\mathrm{working}\,\mathrm{volume}\times \mathrm{VS}\,\mathrm{of}\,\mathrm{inoculum}\,\mathrm{in}\%}{\begin{array}{c} \mathrm{VS}\,\mathrm{of}\,\mathrm{substrate}\,\mathrm{in}\%\times \mathrm{ratio}\,\mathrm{of}\,\mathrm{substrate}\,\mathrm{in}\%\\ \times \mathrm{VS}\,\mathrm{of}\,\mathrm{the}\,\mathrm{sample}\,\mathrm{in}\% \end{array}}$$

**TABLE 2:** Experimental setups for eight different anaerobic digestion reactors.

Reactor type	Inoculum (ml)	Substrate (ml)	Distilled water (ml)
Inoculum	290	0	110
Cellulose	290	1.2 (cellulose)	108.8
Chicken feathers	290	1.2 (feathers)	108.8
Chicken feathers hydrolysate (Microbial pretreated)	290	110 (hydrolysate)	0
Rice husk	290	1.2 (rice husk)	108.8
Rice husk (acid-pretreated)	290	1.2 (rice husk)	108.8
Co-digestion (hydrolysate + rice husk)	290	0.6 (rice husk) + 55 (hydrolysate)	54.4
Co-digestion of hydrolysate with pretreated rice husk)	290	0.6 (rice husk) + 55 (hydrolysate)	54.4

The reactors were flushed with nitrogen and immediately closed with a rubber stopper. The reactors were shaken daily manually.

The pH of all setups was analyzed initially and at the end of the AD. The batch process was continued for 60 days, and the data were recorded until the production of biogas was recoded zero for 5 consecutive days. At the end of the experiment, the accumulated biogas production and biogas yield were compared. To determine the increase in biogas yield due to co-digestion, the biogas yield was compared to the calculated biogas yield (calculated from the mono-digestion of each substrate).

#### Experimental Setup for Continuous Anaerobic Fermentation Reactors

The experimental setup for the continuous process was carried out in a 2.5-L glass reactor for 60 days with a working volume of 2 L. In the current study, three different anaerobic reactors were designed, along with two controls. In the first reactor, mono-digestion of the hydrolysate was carried out. In the second reactor, green waste is fed as a substrate for biogas production. In the third reactor, the feasibility of co-digestion of green waste and microbial pretreated CFs’ hydrolysate was checked. The green waste and hydrolysate were mixed in the ratio of 50:50 on the basis of VSs. The inoculum (2 L) was added to all three reactors, then flushed the headspace with nitrogen to remove the oxygen, and then fixed the glass reactors with cork. The corks were tightly fitted to prevent the passage of air or moisture in or out of the reactor. Two short rods were fixed in the cork; one end of the first rod was inside the glass reactor, and other end was to the outside, to which an airtight bag was attached through a rubber pipe for biogas collection, and the second rod was attached to a 60-ml syringe through a rubber pipe, through which the substrate was introduced, and the same amount of digestate was collected daily at a specific time. The reactors were daily shaken manually.

The hydraulic retention time for continuous anaerobic fermentation of the reactors was 20 days. This time was more than the doubling time of methanogens, and most organic matter could be degraded in respective time. In case of lower retention time, the active cells may wash out from the reactor resulting in lower yield of biogas and could results in process failure (Jeppu et al., 2021). At the same time, to avoid process failure due to overloading, the organic loading rate was kept at 1 g VS/L day, whereas the flow rate was 100 ml/day.

The pH of all the reactors was analyzed daily throughout the incubation. All reactors were kept at 37°C in the incubators.

### Analytical Methods

As discussed previously, the reactor outlet is connected to a gas-tight bag to collect biogas produced in the reactor. After 24 h, the biogas from the bag was measured by a 60-ml syringe. The methane contents of biogas were determined by passing the gas through the scrubbing solution (3 M NaOH). Along with this, volatile fatty acid (VFA) and alkalinity of the effluent were determined with the standard method (American Public Health Association [APHA], 1998b).

### Statistical Analysis

Data were shown as mean ± *SE* for three replicates of each treatment. The significance of difference among different reactors was performed using analysis of variance (ANOVA), and significance of differences among treatment means (Tukey’s test) was performed using the “ggpubr” package in R. The graphs were designed using the “ggplot2” package in R software.

## Results

### Biogas Production, Biogas, and Methane Yield *via* a Batch Process

**FIGURE 1:**
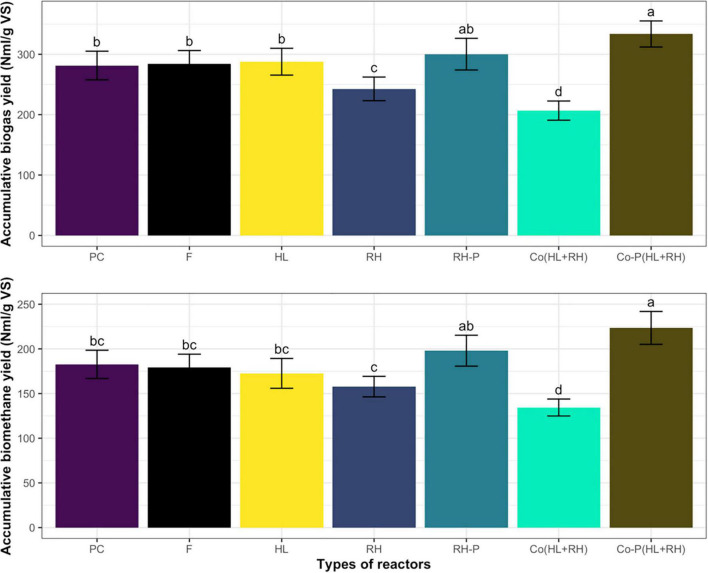
**(A)** Accumulative biogas yield of PC (positive control containing cellulose), F (feathers), HL (feathers hydrolysate), RH (rice husk), RH-P (rice husk pretreated), Co (HL+RH) [co-digestion (hydrolysate + rice husk)], Co-P (HL+RH) [co-digestion with pretreated (hydrolysate + rice husk)]. Means having the same letter are statistically similar as per the Tukey test at *p* < 0.05. **(B)** Accumulative biomethane yield from PC (positive control containing cellulose), F (feathers), HL (feathers hydrolysate), RH (rice husk), RH-P (rice husk pretreated), Co (HL+RH) [co-digestion (hydrolysate + rice husk)], Co-P (HL+RH) [co-digestion with pretreated (hydrolysate + rice husk)]. Means having the same letter are statistically similar as per the Tukey test at *p* < 0.05.

The accumulative biogas and methane yield during the anaerobic batch process by co-digestion of RH-P and feathers hydrolysate Co-P (HL+RH) was higher than all other substrates as represented in Figures 1A,B. The biogas yield was 333.6 N ml/g VS, whereas the methane yield was 223.5 N ml/g VS. Followed by RH-P, the biogas and methane yield was high measured in the case of HL. However, the lowest biogas and methane yield was shown by the co-digestion of hydrolysate and non-pretreated rice husk Co (HL+RH).

**FIGURE 2:**
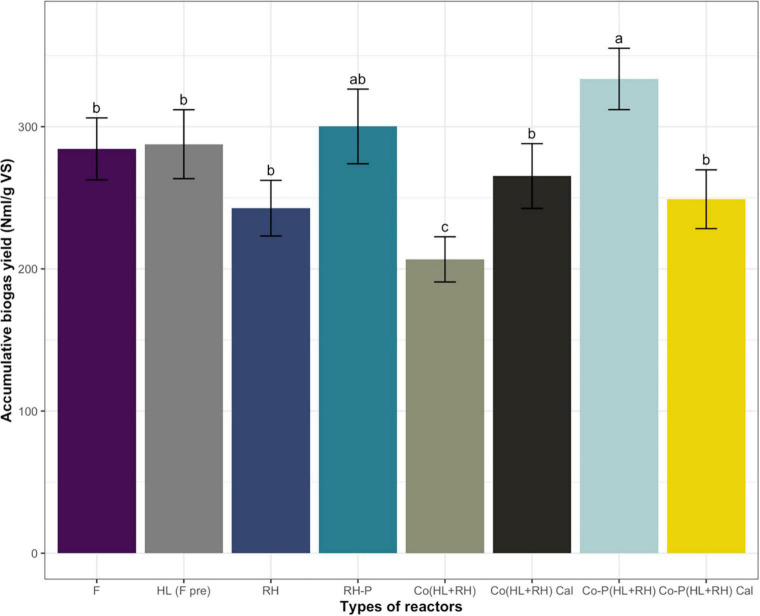
Comparison of accumulative and calculated biogas yield (calculated from mono-digestion of hydrolysate and rice husk) from F (feathers), HL-(F-pre) (feathers hydrolysate biologically pretreated), RH (rice husk), RH-P (rice husk pretreated), Co (HL+RH) [co-digestion (hydrolysate + rice husk)], Co (HL+RH) Cal [co-digestion (hydrolysate + rice husk) calculated from the yield of hydrolysate and rice husk], Co-P (HL+RH) [co-digestion with pretreated (hydrolysate + rice husk)], Co-P (HL+RH) calculated [co-digestion with pretreated (hydrolysate + rice husk) calculated from the yield of hydrolysate and rice husk]. Means having the same letter are statistically similar as per the Tukey test at *p* < 0.05.

In the case of non-pretreated CFs, biogas yield was 284 N ml/g VS, which was increased to 287 N ml/g VS through microbial pretreatment in the hydrolysate. The increase in biogas yield in the case of microbial pretreated CFs was not significant at 1.1%. On the other hand, in the case of non–RH-P, the biogas yield was 242.7 N ml/g VS but increased to 300.2 N ml/g VS in RH-P. The chemical pretreatment of RH significantly increased the biogas yield by 23.75%. To find the increase in biogas yield due to co-digestion, the actual yield from co-digestion was compared to calculated yield for co-digestion (calculated from mono-digestion of hydrolysate and RH). The calculated yield for co-digestion without pretreatment was 265.3 N ml/g VS, which was decreased to 206.7 N ml/g VS in co-digestion. The co-digestion of non–RH-P and CF shows a 20% decrease in methane yield. The calculated biogas yield for pretreated co-digestion was 249 N ml/g VS, which was increased to 333.6 N ml/g VS in the case of Co-P (HL+RH) yield, as represented in Figure 2. In comparison, the co-digestion of hydrolysate with RH-P showed a significant increase of 34% in biogas yield.

#### Process Stability Parameters in Anaerobic Batch Reactors

**FIGURE 3:**
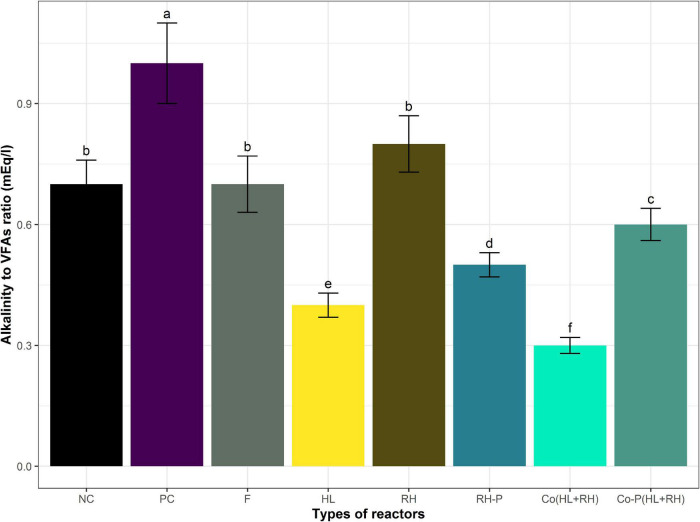
Alkalinity to VFAs ratio of different reactors. Means having the same letter are statistically similar as per the Tukey test at *p* < 0.05.

The pH of all the reactors was analyzed before and after the incubation. The initial pH was set as 7.2. In the case of PC, RH, and RH-P, the pH was slightly decreased to 7.1, 7.14, and 7.08, respectively. However, the pH was still in the optimum range required for AD, whereas in the case of HL, Co (HL+RH), and Co-P (HL+RH), the pH was slightly increased, which are 7.4, 7.6, 7.3, and 7.5, respectively. At the end of the experiment, the VFAs and alkalinity were determined and were found to be in the optimum range. The VFA-to-alkalinity ratio is shown in Figure 3. The highest VFAs accumulation, 875 mg/L, was recorded in hydrolysate followed by Co (HL+RH), 450 mg/l, whereas it was lowest in the negative control.

The lowest 0.3 VFA-to-alkalinity ratio was in Co (HL+RH) followed by 0.4, 0.5, 0.6, 0.7, and 0.8 in feather hydrolysate, RH-P, co-digestion with co-digestion RH-P, negative control, and RH non-pretreated, whereas the PC shows the highest ratio of 1.0.

**FIGURE 4:**
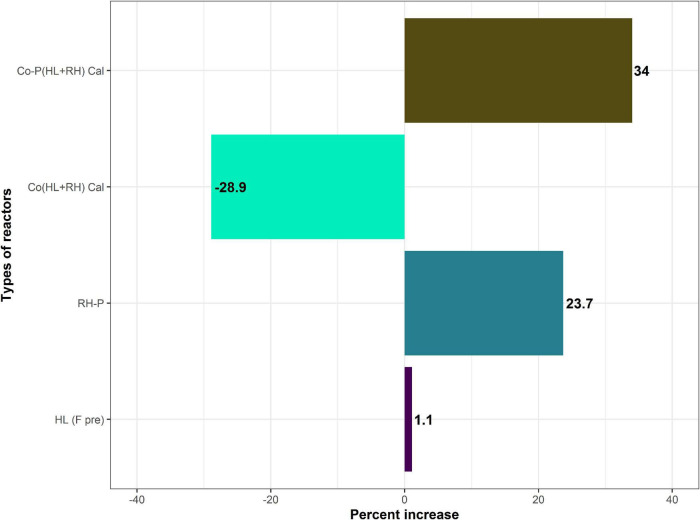
Percent increase in yield of HL (hydrolysate in comparison with feathers), RH-P (rice husk-pretreated in comparison with non-pretreated), and Co (HL+RH) co-digestion (hydrolysate + rice husk in comparison with calculated co-digestion of hydrolysate + rice husk) is decreased, Co-P (hydrolysate + rice husk in comparison with calculated co-digestion of hydrolysate + rice husk).

From the finding during batch process, it is concluded that co-digestion of microbial free treated CFs with the carbon rich RH-P significantly increased the biogas yield. However, when CFs were co-digested with non–RH-P, it did not result in significant increase of biogas yield; therefore, the easily degradable carbon rich green waste was used in the continuous process as represented in Figure 4.

### Continuous Anaerobic Fermentation

The hydrolysate was co-digested with GW during continuous AD to enhance biogas production and process stability. The co-digestion significantly increased the biogas yield, process stability, and VS removal in a continuous process.

#### Biogas and Biomethane Yield

**FIGURE 5:**
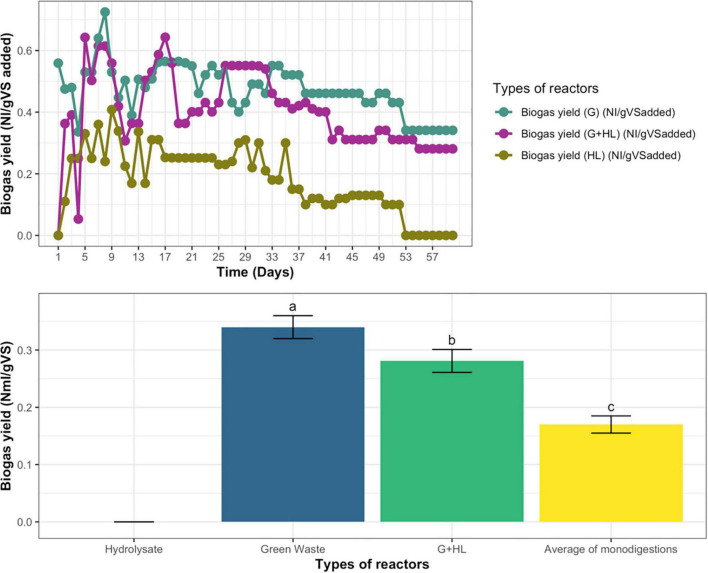
**(A)** Daily Biogas yield of hydrolysate (HL), green waste (G), and their co-digestion (G+H). **(B)** Biogas yield of co-digestion compared with an average of mono-digestions for continuous anaerobic fermentation. Means having the same letter are statistically similar as per the Tukey test at *p* < 0.05.

Biogas yield of continuous anaerobic mono-digestion of microbial pretreated CFs’ hydrolysate (HL), GW, and their co-digestion (G+HL) for three retention times was determined. Daily biogas production was measured as normalized liters. The biogas yield was fluctuating daily with time initially, but, at steady-state, the biogas yield for microbial pretreated CFs’ hydrolysate (HL) becomes zero, GW become 0.34 N L/g VS added, whereas, for co-digestion (G+HL), the biogas yield become 0.281 N L/g VS added, as described in Figure 5A.

The biogas yield of co-digestion was compared with the average values of both the mono-digestions, which indicated that their average was less than the biogas yield of co-digestion as represented in Figure 5B.

**FIGURE 6:**
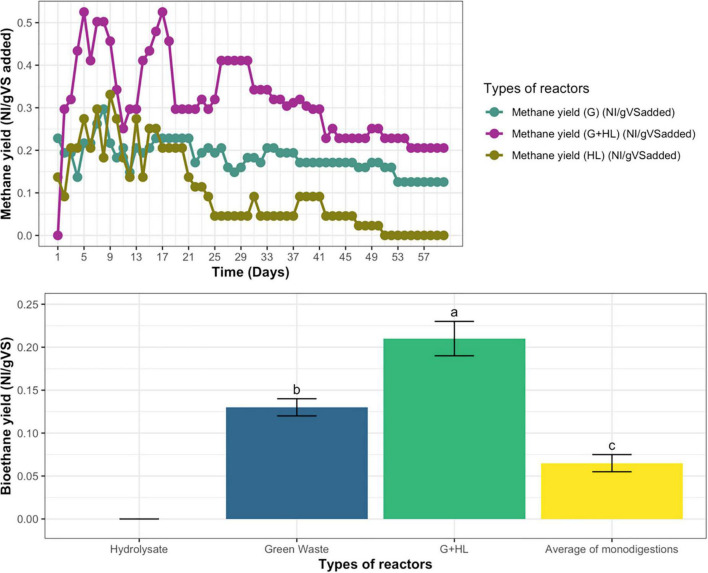
**(A)** Daily biomethane yield of hydrolysate (HL), green waste (G), and their co-digestion (G+H). **(B)** Biomethane yield of co-digestion compared with an average of mono-digestions for continuous anaerobic fermentation. Means having the same letter are statistically similar as per the Tukey test at *p* < 0.05.

The biomethane was measured for all the continuous anaerobic reactors; at the beginning, the biomethane fluctuated with time, but, at steady-state, the biomethane for microbial pretreated CFs’ hydrolysate (HL) reactor becomes zero, GW becomes 0.13 N L/g VS added, whereas, in the case of co-digestion on addition of (G+HL), it becomes 0.21 NL/g VS, as represented in Figure 6A.

Whereas, the biomethane yield of co-digestion was compared with the average values of both the mono-digestions, which indicated that their average was less than the biomethane yield of co-digestion (G+HL), as represented in Figure 6B.

#### Process Stability

The pH, VFAs, and alkalinity were used as parameters to monitor the stability of the process. The volatile fatty acids and alkalinity were measured for all the anaerobic reactors represented in Table 3. Initially, the pH of all reactors was adjusted to 7.2, which is optimal for biogas production. During steady-state, the pH of hydrolysate increased to 8.5; in the case of green waste, the pH decreased to 6.8; whereas, in the case of co-digestion, no significant change in pH was recorded. The VFA accumulation was 6,250, 5,000, and 5,750 mg/L in the case of mono-digestion of hydrolysate, mono-digestion of green waste, and co-digestion of hydrolysate and green waste, respectively. Whereas, the VFA-to-alkalinity ratio was 0.7, 0.8, and 0.9 in the case of mono-digestion of hydrolysate, mono-digestion of GW, and co-digestion of hydrolysate and green waste, respectively.

**TABLE 3:** Process stability parameters during the continuous anaerobic digestion process.

Reactor type	pH	VFAs (mg/L)	Alkalinity (mg/L)	VFA-to-alkalinity ratio
Mono-digestion of hydrolysate	8.5	6,250	9,000	0.7
Mono-digestion of green waste	6.8	5,000	6,125	0.8
Co-digestion of hydrolysate and green waste	7.1	5,750	6,250	0.9

*Means having the same letter are statistically similar as per the Tukey test at p < 0.05.*

## Discussion

The expanding interest toward biogas generation from bio-waste through complex AD opened new routes in improving biogas production processes and their upgradation. Waste generation and waste handling are the most critical issues to overcome these days. CF is one of the major waste, generated in a considerable amount. Feathers can be utilized for energy production by eliminating harmful environmental and catastrophic issues. In this way, on one hand, waste is properly managed, and, on the other hand, bioenergy in the form of biogas is produced (Provin et al., 2021) from the waste. Therefore, the current study was designed to evaluate the effect of different pretreatments and co-digestion and mono-digestion by AD using batch and continuous fermentation and its effect on biogas yield. The VSs of CFs, feathers hydrolysate, and RH were 70, 1.05, and 75.98%, respectively. VS depends upon the type, source, and composition of the substrate.

The results of accumulative biogas production demonstrated that, in the case of PC, most of the gas was produced in the initial 15 days of the experiment, whereas, in the case of RH pretreated co-digestion, it took 30 days. In the case of non-pretreated co-digestion, RH, and pretreated rice, there was a gradual increase in production until the end. On the other hand, in the case of feathers, the production was low until 25 days, and, after that, it was increased to reach the maximum level. The time taken by the process depends upon the substrate composition, whereas the PC contains glucose that was rapidly utilized by acidogens. Different research results reported that hydrolysis depends on substrate composition and carbohydrate hydrolyzed in hours, whereas complex compounds like cellulose and lignin took weeks to hydrolyze completely. Some other studies reported that some substrate has hardly accessible for the action of enzymes that ultimately took more time, and the hydrolysis became a rate-limiting step. The present study demonstrated (Figures 1A,B) that the microbial pretreatment was completed in 72 h of incubation. According to the research work reported by Forgács et al. (2011), with different incubation times of 1, 2, and 8 days, the biogas yield was 55, 94, and 83%, respectively. The possible reason was the VFA accumulation in the hydrolysate at the end of the experiment as the VFA measured in the hydrolysate was 875 mg/L and, in untreated feathers, was 275 mg/L. The VFA accumulation in microbial pretreated CFs’ hydrolysate was expected due to the accumulation of ammonia, which negatively affected the methanogenic population. Ammonia is linked to the presence of protein sources in the feed. Because CFs are mainly composed of protein, hydrolysis results in ammonia accumulation (Sypka et al., 2021). During AD of the nitrogenous feedstock, ammonia inhibition is one of the main challenges, resulting in lower biogas yield (Chen et al., 2008). At lower concentrations, ammonia acts as a buffering agent, but, in a low C/N ratio, the accumulation of ammonia negatively affects the methanogenic population; hence, the nitrogenous feedstock must be co-digested with the carbon-rich substrate (Christou et al., 2021). Figure 1A represents the accumulative biogas yield; the microbial pretreated feathers slightly increased the biogas yield by 1.1% compared to non-pretreated feathers. The results followed the study reported by Forgács et al. (2011) that determined the effect of microbial pretreatment using different wild and recombinant bacterial strains from CFs. Another study also reported that there was no significant increase in methane yield of CFs when biological treated with *Bacillus megaterium*. However, the analysis of hydrolysate showed that the total dissolved solids were increased by 1%. At the end of the incubation, VFA accumulation in the pretreated feathers was 875 mg/L, whereas, in non-pretreated, it was 275 mg/L. This clearly indicates that the microbial pretreatment increases the feathers’ hydrolysis but results in low biogas yield due to ammonia inhibition. In the case of the reactor fed with non–RH-P, accumulative biogas yield was lower than the RH-P and CFs (Figure 2). The chemical pretreatment of RH increases the biogas yield compared to non–RH-P. Different research results reported increased biogas yield due to acidic pretreatment at different concentrations and conditions. According to the result reported by He et al. (2017), the pretreatment of RH with 6% NaOH resulted in the biogas yield of 0.53N L/g VS, whereas non-pretreatment can yield 0.36 N L/g VS. The chemical pretreatment of RH showed a 32% increase in biogas yield compared to untreated RH in the case of mono-digestion. Mu et al. (2021) also checked the effect of acid mixtures like acetic and propionic acid but do not measure any variance in the treated and untreated RH. In another study by Dahunsi (2019), the pretreatment with 2% sulfuric acid may reduce the hemicellulose content by 69%. The possible reason was that the chemical pretreatment improves biodegradability, increases the biogas yield, and enhances the efficiency of the overall process. The biomass structure could be changed through chemical pretreatment methods to have more accessibility to anaerobic microorganisms, therefore enhancing the biogas production and digestion efficiency. At the end of the experiment, the VFA accumulation was 250 and 350 mg/L in RH and RH-P, respectively, which indicates the increase in hydrolysis due to pretreatment.

During the current study, the effect of co-digestion on biogas yield with pretreated and non–RH-P was determined and compared with the calculated methane yield for RH pretreated co-digestion and RH non-pretreated co-digestion. In the case of non-pretreated co-digestion, the calculated yield was determined from the yield of mono-digestion of non–RH-P and yield of hydrolysate. In the case of non-pretreated co-digestion, the accumulative biogas yield and methane yield were lower than the calculated methane yield. In the co-digestion in the case of non–RH-P, the methane yield was 22.2% decreased. The decrease in the case of non–RH-P with cattle manure is supported by Lange et al. (2016). This decrease in yield may be due to ammonia accumulation, as the feathers have high protein contents (Mohanty et al., 2021). Furthermore, RH has a complex lignocellulosic structure that is resistant to degradation. At the same time, CFs have a high concentration of proteins, whereas, during degradation of feathers, ammonia is produced due to microbial pretreatment. Ammonia is very toxic in higher concentrations (Christou et al., 2021), and at the end of the incubation, VFA accumulation in the hydrolysate was 875 mg/L, whereas, in RH, it was 250 mg/L. In the case of co-digestion, VFAs were lower than hydrolysate, which confirms that the ammonia in co-digestion affects the hydrolysis of RH, which was the reason for a lower yield in co-digestion as compared to the calculated yield (Figure 3). Magrí et al. (2017) reported that the yield of biogas depends upon the composition and characterization of the substrate. Nakamura and Asada (2021) reported that RH contains high cellulose and lignin, resistant to enzymatic degradation, leading to low biogas yield. However, the increase in biogas yield due to co-digestion depends upon the co-substrate and pretreatment. Hamed et al. (2015) reported a significant increase in biogas yield resulting from mixing cattle manure with food waste in different ratios.

According to the current research work, the methane yield from co-digestion of hydrolysate with pretreated RH was compared to calculated methane yield compared to the yield of mono-digestion of hydrolysate and yield of mono-digestion of RH-P. The co-digestion of hydrolysate with pretreated RH increases the biogas yield by 34% compared to the calculated yield as represented in Figure 4. Different studies have reported an increase in biogas yield in the case of co-digestion. Sayara and Sánchez (2019) reported a 758% increase in biogas yield in the case of co-digestion of cattle manure with pretreated RH. Dahunsi (2019) reported a 52% increase in biogas yield in co-digestion of cattle manure with cocoa husk. The increase in biogas yield was due to the pretreated RH, which has more sugar than non-pretreated. It was reported previously that chemical pretreatment improves biodegradability and increases the biogas yield and enhance efficiency of the overall process (Khan and Ahring, 2021). The biomass structure could be changed through chemical pretreatment methods to have more accessibility to anaerobic microorganisms, therefore enhancing the biogas production and digestion efficiency. The other reason for an increase in biogas production may be the effect of a more balance C/N ratio in the case of co-digestion of hydrolysate and RH-P. The nutrients and organic content present in the feedstock affect the activity and growth of the microorganisms. The essential nutrients for microorganisms are carbon and nitrogen (Mothe and Polisetty, 2021). The deficiency of any nutrient ceases the growth and activity of the microorganism. A low C/N ratio results in the formation of ammonia, whereas a high ratio results in the failure of the biogas plant. Hence, in both cases, AD failed (Nkuna et al., 2021). Appropriate C/N ratio/nutrient balance is required for the proper growth of microorganisms under stable environmental conditions. Furthermore, this is why the C/N ratio greatly influences AD (Xiao et al., 2021). Generally, the most appropriate (C/N) ratio of 25–30 was required for the suitable development of biological processes (Ramírez-Sáenz et al., 2009; Liu et al., 2016; Sillero et al., 2021). Methane production decreases because of the imbalance in the C/N ratio. Moreover, the most common problem was the inhibition by nitrogen in degrading organic wastes. Hence, the problem of the C/N ratio could be overcome by co-digestion with a waste having a high C/N ratio. At the end of the experiment, the VFA accumulation in co-digestion pretreated RH and hydrolysate was lower than both mono-digestion of hydrolysate and pretreated RHs in Figure 3. The lower VFA accumulation in the co-digestion of hydrolysate and pretreated RH means that most of the VFAs are converted to biogas, leading to high yield.

During continuous AD, biogas and methane yield for mono-digestion of microbial pretreated CFs’ hydrolysate (HL), GW, and their co-digestion (G+HL) was measured for three retention times. The biogas yield fluctuated daily with time initially, but, at steady-state, the biogas yield for microbial pretreated CFs’ hydrolysate (HL) becomes stable, as shown in Figures 5A,B. In the case of mono-digestion of hydrolysate, the biogas yield showed a continuous decline with time, leading to process failure in the third retention time. The decline in biogas yield and process failure was due to ammonia accumulation as a result of the high protein content of feathers (Mohanty et al., 2021), as, during degradation of feathers, ammonia is produced due to microbial pretreatment. Ammonia was toxic in higher concentrations (Christou et al., 2021). On the other hand, the biogas yield on green waste and co-digestion was more stable during steady-state. As a consequence of co-digestion, the biogas and biomethane yield increased compared to the average of GW and hydrolysate mono-digestions, as shown in Figures 5, 6. Co-digestion with substrates with high buffering capacity (alkalinity) such as manure can be good alternatives for the effective treatment of highly biodegradable materials. During the co-digestion of microbial pretreated CF hydrolysate with GW and animal manure, the manure provides buffering capacity and various nutrients. In contrast, the plant material provides high carbon content (Ambaye et al., 2021), resulting in a high biogas yield. The result is a more balanced C/N ratio, and the co-digestion of manure and green-grocery waste also decreases the risk of ammonia inhibition and acidification. Thus, it was better to consume microbial pretreated CF hydrolysate in co-digestion than mono-digestion for biogas production. GW has a high content of carbon and feather hydrolysate has a high amount of nitrogen, so when they both were used in co-digester to balance the C/N ratio and produce more biogas and biomethane as compared to the average of their mono-digesters, thus enhancing the biogas yield and the efficiency of overall process (Kiran et al., 2016).

Furthermore, at the start of the experiment, the pH of all the reactors was set to be 7.2, whereas, at the end of the experiment, the pH of hydrolysate increased. In the case of GW, the pH was dropped down to a critical level, whereas, in the case of co-digestion, there was no significant change in pH. The pH range varies for different groups of microorganisms for their growth, like for the growth of fermentative bacteria, pH was required about 4.0–8.5, and, for methanogenic bacteria, the pH must be in the range of 6.8–7.2; otherwise, failure of the process may occur (Amin et al., 2021). On higher pH levels, the free ammonia concentration increased, causing more toxicity for methanogens (Wang et al., 2020). The VFA accumulation was high in the case of hydrolysate and finally led to process failure. Whereas, in the case of co-digestion, the VFA accumulation was lower than mono-digestion of hydrolysate and green waste. The co-digestion enhances the process stability and reduces the toxic effect of ammonia, resulting in an increase in biogas production (Christou et al., 2021).

## Conclusion

Non-pretreated CFs were challenging to hydrolyze by anaerobic microorganisms because of their recalcitrant nature. Microbial pretreatment of CFs by *Pseudomonas aeruginosa* increased its hydrolysis, but the mono-digestion of hydrolysate results in low yield of biogas due to high concentration of ammonia. On the other hand, RH’s pretreatment significantly increased biogas yield. Pretreated RH co-digestion with hydrolysate significantly increased biogas production. However, co-digestion with non–RH-P does not enhance the biogas yield. During continuous AD, the hydrolysate’s co-digestion with GW significantly increases the biogas yield. These results suggest the importance of running the bioreactor for a long term in continuous fermentation mode to test the suitability of a novel biomass substrate for industrial biogas production.

## Data Availability Statement

The original contributions presented in the study are included in the article/supplementary material, further inquiries can be directed to the corresponding author/s.

## Author Contributions

MS, AnK, HA, AB, ZG, AlK, and MMR contributed to design of the study, performed the experiments, and wrote the first draft of the manuscript. MB, FH, and AS wrote the comments, review, and involved in supervision of the students. SK was the main supervisor and secured funding. All authors contributed to manuscript revision, read, and approved the submitted version.

## Conflict of Interest

The authors declare that the research was conducted in the absence of any commercial or financial relationships that could be construed as a potential conflict of interest.

## Publisher’s Note

All claims expressed in this article are solely those of the authors and do not necessarily represent those of their affiliated organizations, or those of the publisher, the editors and the reviewers. Any product that may be evaluated in this article, or claim that may be made by its manufacturer, is not guaranteed or endorsed by the publisher.
